# A High Cattle-Grazing Density Alters Circadian Rhythmicity of Temperature, Heart Rate, and Activity as Measured by Implantable Bio-Loggers

**DOI:** 10.3389/fphys.2021.707222

**Published:** 2021-08-13

**Authors:** Carlos Palacios, Javier Plaza, José-Alfonso Abecia

**Affiliations:** ^1^Departamento de Construcción y Agronomía, Facultad de Ciencias Agrarias y Ambientales, Universidad de Salamanca, Salamanca, Spain; ^2^Departamento de Producción Animal y Ciencia de los Alimentos, Instituto Universitario de Investigación en Ciencias Ambientales (IUCA), Universidad de Zaragoza, Zaragoza, Spain

**Keywords:** cattle, density, bio-loggers, circadian rhythm, temperature, heart rate, activity

## Abstract

Six cows managed under extensive grazing conditions were used to study the effect of moving the animals to a higher grazing density on the circadian rhythms of temperature (T), heart rate (HR), and activity (ACT), which were recorded by implantable bio-loggers. Cows were maintained at a density of 1.5 livestock units per hectare (LSUs/ha; low density, LD) until they were moved to a grazing area at 128 LSUs/ha (high density, HD). Animals were implanted subcutaneously with a T, HR, and ACT bio-logger, which was programmed to record data at 5-min intervals. For each animal, cosinor rhythmometry (the study of circadian rhythms by fitting a sine wave to a time series) was applied to the data recorded over 5 days in LD and HD. Mean Midline Estimating Statistic of Rhythm (MESOR; the average value around which the variable oscillates), amplitude (difference between the peak and the mean value of a wave), and acrophase (timing of peak activity) were calculated and evaluated statistically. Differences between mean day and nighttime values, and mean LD and HD values were calculated. Cows presented cosinor curves that fit a 24-h rhythm (*p* < 0.001) in T, HR, and ACT at both densities. MESOR (T: 37.98 vs. 38.02°C; HR: 69.12 vs. 65.91 bpm; ACT: 49.39 vs. 40.41 mg, for LD and HD, respectively) and amplitude (T: 0.28 vs. 0.28°C; HR: 4.12 vs. 3.14 bpm; ACT: 18.14 vs. 11.28 mg, respectively) did not differ significantly between the two densities; however, significant (*p* < 0.05) differences between densities occurred in the acrophase of the three variables; specifically, the T acrophase was 2 h later at HD (22:45 h) than LD (20:45 h), and HR (LD: 19:51; HD: 16:49 h) and ACT acrophases 3 and 2 h earlier at HD than LD (LD: 14:47; HD: 12:49 h), respectively. T and ACT differed significantly (*p* < 0.01) between daytime (mean ± SE; 37.92 ± 0.19°C, 40.39 ± 4.74 mg) and nighttime (38.14 ± 0.17°C, 29.93 ± 5.66 mg). In conclusion, our study suggests that a high animal grazing density might exacerbate the social competence for valuable resources for animals, resulting in shifting the circadian rhythmicity of temperature, heart rate, and activity of the cows, advancing or delaying their acrophases.

## Introduction

Environmental factors such as temperature (T), humidity, and solar radiation have significant direct and indirect effects on livestock production (Nienaber et al., 1999). Climate change has been generating temperatures that negatively affect the availability of food for animals maintained in grazing systems. Heat and nutritional stress are the most influential factors in the productive and reproductive successes in cattle (Maurya et al., 2004; Marai et al., 2007). Different production practices, some of which involve high animal density and rotational grazing in order to improve soil conditions and the yield and quality of forage (Murphy et al., 1986) have been implemented. Organic livestock production involves the environmentally sustainable animal management, with animal welfare as a priority, and with a high consumer demand (Von Borell and Sørensen, 2004). Stocking density is one of the factors that can produce stress in the animals (Loudon et al., 2019) and organic management might be a means of preventing this stress, through its specific legislation regarding low animal densities.

To collect information that indicates the level of stress suffered by the animal, there are animal-attached devices that record body temperature, heart rate (HR), and activity (ACT) of an individual. For instance, HR measures the activity of the autonomous nervous system (Sanmiguel et al., 2018), commonly used in animal welfare research to measure changes in the sympathetic-vagal balance in mammals and birds in relation to the circadian rhythm (Brauner et al., 2010), management, health, psychological, and environmental stressors (Stubsjoen et al., 2015). The stress-induced hyperthermia response is a relatively short-lasting rise in body temperature in response to stress (Bouwknecht et al., 2007). Stress-induced pathologies can be accompanied by changes in circadian rhythms; therefore, a study of these changes might be helpful in confirming whether a group of animals is under a stressor that ensures animal welfare. Temporal evaluation of a fluctuating variable that has a rhythmic variation will provide a forecast for making decisions, because biological rhythms and the state of the health of an animal and a population are correlated (Piccione and Caola, 2002).

In recent years, the development of bio-sensors for monitoring real-time responses in, for example, body temperature, respiration and heart rate, blood pressure, or activity of the animals, have helped to understand how external factors (e.g., housing, diet, and management) affect an animal’s resiliency to stressors (Neethirajan et al., 2017). Data recorded by these devices can be analyzed by cosinor analysis, which is often used in the analysis of biological time series that demonstrate predictable rhythms fitting a sine wave to a time series. Since it has been observed that changes in feeding time, housing procedures, or social cues can affect biological rhythms in animal experiments (Piccione and Caola, 2002), we hypothesize that changes in animal density of grazing cows could lead to a modification of their circadian rhythmicity.

The objective of this study was to use subcutaneous bio-loggers to investigate the circadian changes in some physiological variables of cows that have been subjected to rotational grazing with a high livestock density in a short period of time.

## Materials and Methods

The Ethics Committee for Animal Experiments at the University of Salamanca approved the procedures performed in this study (reference PI/2020/956). The care and use of animals were in accordance with the Spanish Policy for Animal Protection (RD 53/2013), which meets the European Union Directive 2010/63 on the protection of animals used for experimental and other scientific purposes.

### Animals

The study was performed on an organic agricultural and livestock farm in Larrodrigo, Spain (40° 44' N; 5° 26' W). The farm was in an area where cereal fields are interspersed among cultivated pastures and holm oak trees. The main cultivated species were forage species that were destined for consumption by the cattle herd. The cattle herd was divided into large grazing groups that grazed fenced pastures that had holm oak, although there were areas of meadows with grasslands. In general, the stocking density in the extensive management was low, although it varied based on the size of the enclosure in which they had been allocated. Animals were always kept outdoors under natural photoperiodic conditions (15 June: sunrise 0650 h, sunset 21:15 h), and water was available ad libitum.

In early April, a group of 90 Morucha × Charolais cows, which had a mean live weight (±SD) of 550 ± 22 kg, was allocated to a fenced grazing area that provided 1.5 livestock units per hectare (LSU/ha; low density, LD). In early June, six cows were selected to be implanted with bio-loggers. They were selected based on their age (5–6 years-old) and homogenous liveweight (540 ± 10 kg). After 1 week, the herd was moved to a smaller grazing area where the density was 128 LSUs/ha (high density, HD). Both areas were adjacent, so they shared similar characteristics of orography, insolation, and vegetation.

### Measurements

Six cows were surgically implanted with a subcutaneous T, HR, and ACT bio-logger (DST centi-HRT ACT, Star Oddi, Gardabaer, Iceland), which was programmed to record data at 5-min intervals. After the experiment, the bio-loggers were removed and the data were downloaded using a communication box and Mercury software v5.83 (Star Oddi, Gardabaer, Iceland). The data were recorded over 5 days in LD after the logger had been implanted and the first 5 days in HD (the transition day was excluded). The DST activity loggers measure acceleration in three dimensions, in relation to earth‘s gravity field. Each logger is calibrated by Star-Oddi, and static acceleration for each axis and then calculated using the following steps. First, the center of the range of values for each axis is measured and extracted from the raw accelerometer value, then each axis is normalized to 1 g based on the range measured for each axis during rotation (g is the acceleration of gravity or 9.8 m s^−2^). The algorithm then uses this calibration information to separate the static and dynamic acceleration components of each measurement. The algorithm calculates the external acceleration (EA) as a vectorial sum of dynamic body acceleration (veDBA; reported by the software in mg), which has been correlated with HR in cattle (Miwa et al., 2015). Mean, maximum, and minimum ambient T and relative humidity (RH, %) on the days of the experiment were collected from the meteorological station on the farm.

### Surgical Implantation

For implantation, the cows were immobilized in the handling race, with the head held and the left leg of the cow extended to reach the armpit. Cows were sedated with 0.05 mg/kg xylazine i.m. (Xilagesic, Calier, Barcelona, Spain), the skin was cleaned for surgery, and a 2-ml local anesthetic s.c. (lidocaine hydrochloride, Anesvet, Ovejero, León, Spain) was injected. An incision was made in the skin, and a pocket to hold the sensor was created. The bio-logger was placed subcutaneous on the left thorax, above the heart, and with the sensor axis parallel to the heart axis. The incision was closed by 2–3 sutures of surgery silk and was sprayed with aluminum spray (Aluspray, Vetoquinol, Madrid, Spain). The bio-loggers were sterilized by a 24-h immersion in 0.55% ortho-phthalaldehyde (CIDEX-OPA solution, Johnson & Johnson, New Jersey, United States). Similar procedures were performed for the sensor withdrawal.

### Statistical Analysis

To test the significance of the differences in T, HR, and ACT between the two grazing densities and between day (07:00–21:00 h) and night (22:00–06:00 h), we used the SPSS MIXED procedure (IBM Corp, 2019), in a model that included grazing density (LD or HD) and day/night time as fixed effects, and their interaction, with cows considered as random effects. Since correlation between residuals of the observations (autocorrelation) is assumed, a first-order autoregressive (AR1) covariance structure was used, so that, the variance is assumed to be heterogeneous and the correlations between the two adjacent time points decline across measurement occasions. The 5-min observations records of each variable were grouped hourly, and recoded for day and night times. Circadian rhythms in T, HR, and ACT were graphed by fitting the time series measurements of each cow to the cosine curve of a 24-h activity rhythm, which was obtained by the cosinor method at the Cosinor on-line platform (Molcan, 2019).^1^ Midline Estimating Statistic of Rhythm (MESOR, the average value around which the variable oscillates), amplitude (the difference between the peak and the mean value of a wave), and acrophase (the time of peak activity) were calculated for each variable in each individual. Cosine curves are y_t_ = M + A cos((2π/24)t + φ) + ϵ_t_, where y_t_ is an observation at time t, M is the MESOR, A is the amplitude, and φ is the acrophase. The error term ϵ_t_ is assumed to be independently normally distributed with mean 0 and unknown constant variance σ2. To test for rhythmicity, an F-test compared the (re-parameterized) cosine model with the nonrhythmic model yt = M + ϵ_t_. A *p* < 0.05 indicates that the time series fit a 24-h rhythm. Thereafter, the data were pooled and the mean 24-h cosinor curve for each of the three parameters was calculated, and the cosinor values of the two densities were compared by an ANOVA. The results were expressed as mean ± SE. Values of *p* < 0.05 were considered statistically significant.

## Results

**Figure 1:**
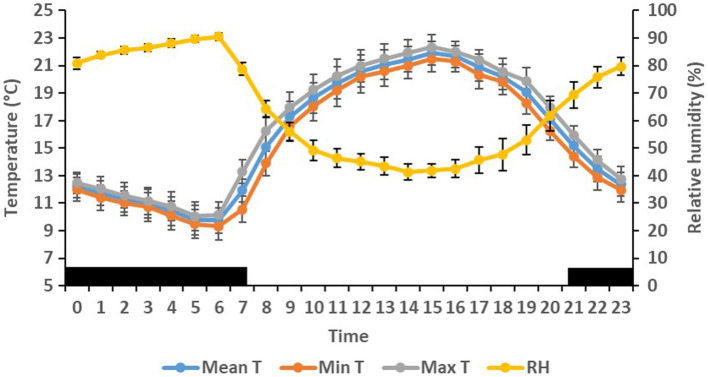
Mean (±SEM) maximum (Max), minimum (Min), mean ambient temperature (T, °C), and relative humidity (RH, %) on the farm in which six cows were exposed to low and high density grazing.

**Figure 2:**
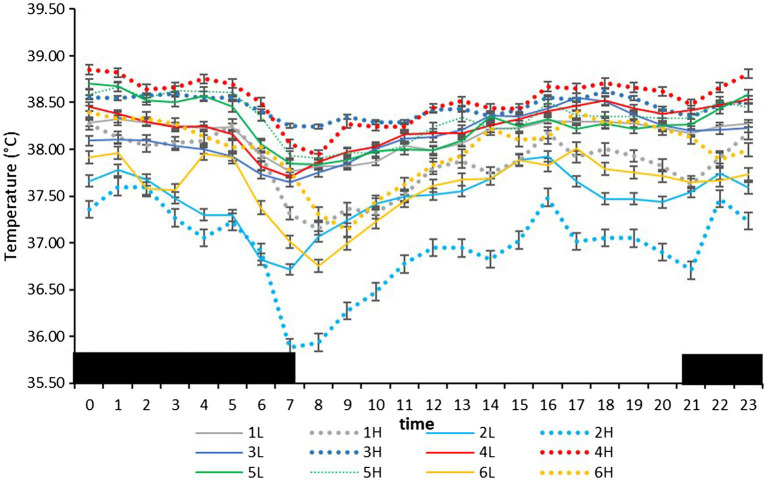
Mean (±SEM) body temperature (°C) of six cows measured by subcutaneous bio-loggers on 5 days of grazing at 1.5 LSUs/ha (low density, l) followed by 5 days of grazing at a density of 128 LSUs/ha (high density, H). Black areas indicate night (22:00–06:00 h).

**Figure 3:**
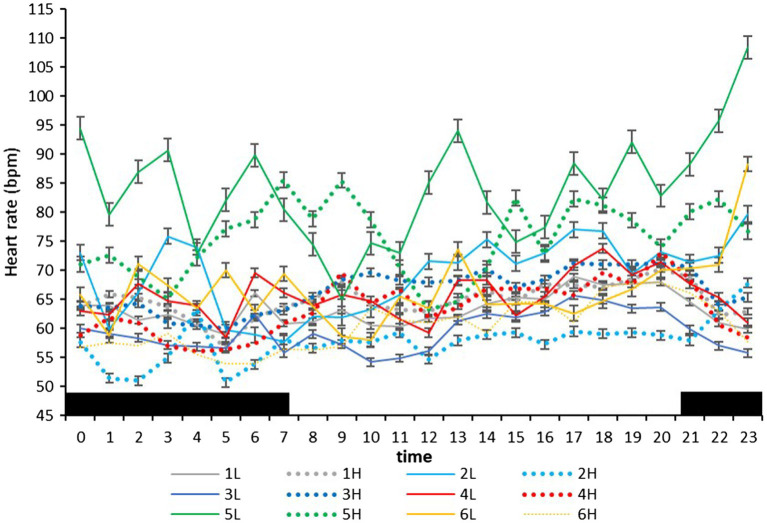
Mean (±SEM) heart rate (bpm) of six cows measured by subcutaneous bio-loggers on 5 days of grazing at 1.5 LSUs/ha (low density, L) followed by 5 days of grazing at a density of 128 LSUs/ha (high density, H). Black areas indicate night (22:00–06:00 h).

**Figure 4:**
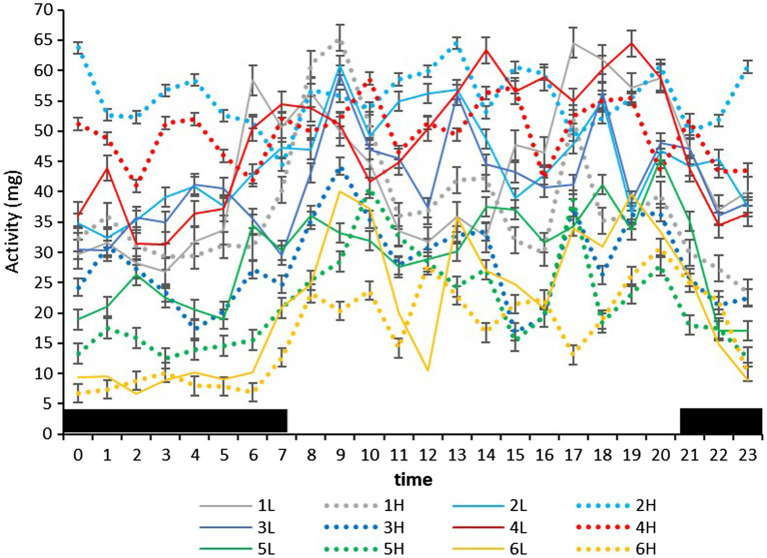
Mean (±SEM) activity (standard acceleration of gravity, *g*) of six cows measured by subcutaneous bio-loggers on 5 days of grazing at 1.5 LSUs/ha (low density, L) followed by 5 days of grazing at a density of 128 LSUs/ha (high density, H). Black areas indicate night (22:00–06:00 h).

**Figure 5:**
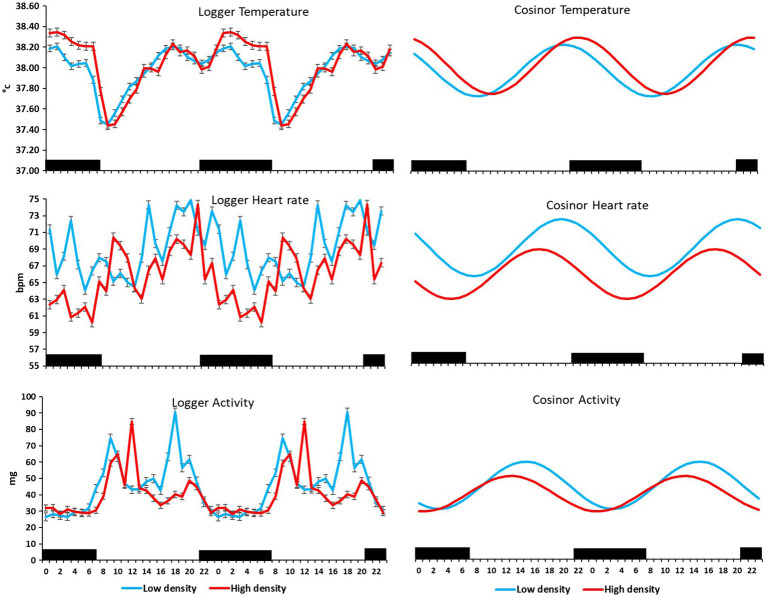
Mean (±SEM) temperature (°C), heart rate (bpm), and activity (standard acceleration of gravity, *g*) measured by subcutaneous bio-loggers **(left panel)**, and the cosine curve of a 24-h activity rhythm **(right panel)** of six cows on 5 days grazing at 1.5 LSUs/ha (low density), followed by 5 days of grazing at a density of 128 LSUs/ha (high density). Black areas indicate night (22:00–06:00 h).

On the farm at the time of the experiment, maximum T and lowest RH occurred in the midday (Figure 1). Temperature and ACT were significantly (*p* < 0.01) affected by light/dark period of the day, but HR was significantly (*p* < 0.001) affected by grazing density, with a significant interaction between density and light/dark period (*p* < 0.001; Table 1). Mean day and night values of T, HR, and ACT under both grazing densities are presented in Table 2. No differences between mean T at LD (37.98 ± 0.01°C) and HD (38.02 ± 0.04°C) were observed. HR was significantly (*p* < 0.001) higher at LD than at HD (67.72 ± 0.32 vs. 64.61 ± 0.20 bpm), and ACT during daytime (40.39 ± 4.74 mg) was significantly higher (*p* < 0.001) than at nighttime (29.93 ± 5.66 mg). The mean values of a 24-h period of the three variables in the six cows at the two densities are presented in Figures 2–4. Cows experienced a dramatic reduction in T at the beginning of the day (Figure 5), which coincided with increases in HR and ACT. Cows exhibited the least movement at night.

**Table 1:** Matrix of coefficients of the mixed model applied to test the significance of the differences in temperature (T), heart rate (HR), and activity (ACT) of six cows that were maintained at 1.5 livestock units per hectare (LSUs/ha; low density, LD) and were moved to a grazing area that had a density of 128 LSUs/ha (high density, HD).

	Value	SE	*t*-value	*p*-value
Temperature				
Intercept	37.490	31.226	1.200	0.229
Density	0.038	0.037	1.019	0.308
Day/Night	−0.037	0.012	−3.098	0.002
Interaction	0.003	0.016	0.176	0.860
Heart Rate				
Intercept	66.711	0.834	79.957	0.000
Density	−5.637	0.806	−6.993	0.000
Day/Night	0.324	0.770	0.402	0.674
Interaction	4.033	1.066	3.78	0.000
Activity				
Intercept	33.621	3.950	8.511	0.000
Density	0.286	3.812	0.075	0.940
Day/Night	24.669	3.638	6.780	0.000
Interaction	−9.530	5.036	−1.892	0.058

**Table 2:** Mean (±SEM) daytime (07:00–21:00 h) and nighttime (22:00–06:00 h) temperature (T, °C), heart rate (HR, bpm), and activity (ACT, mg) of six cows that were maintained at 1.5 LSUs/ha (LD) and were moved to a grazing area that had a density of 128 LSUs/ha (HD).

	T		HR		ACT	
	LD (*n* = 6)	HD (*n* = 6)	LD (*n* = 6)	HD (*n* = 6)	LD (*n* = 6)	HD (*n* = 6)
Daytime	37.93 ± 0.01^x^	37.90 ± 0.01^x^	67.63 ± 0.38^a^	66.34 ± 0.25^b^	43.13 ± 0.65^x^	37.74 ± 0.57^x^
Nighttime	38.04 ± 0.01^y^	38.23 ± 0.01^y^	67.87 ± 0.58^a^	61.71 ± 0.33^b^	29.55 ± 0.42^y^	30.17 ± 0.43^y^

a, b indicate *p* < 0.05 between densities; x, y indicate *p* < 0.05 between day and night.

The six cows had cosinor curves that fitted a 24-h rhythm (*p* < 0.0001) in T, HR, and ACT at both grazing densities (Figure 5). MESOR and amplitude of the three variables did not differ significantly between the two densities (Table 3); however, the acrophase of T, HR, and ACT differed significantly (*p* < 0.05) between densities, such that the time of peak T was 2 h later at HD than it was at LD, and peak HR and ACT were 3 and 2 h, respectively, earlier at HD than they were at LD (Table 3).

**Table 3:** Mean (±SEM) Midline Estimating Statistic of Rhythm (MESOR), amplitude (the difference between the peak and the mean value of a wave), and acrophase (the time of peak activity) of the cosine curve of a 24-h activity rhythm in temperature (°C), heart rate (bpm), and activity (mg), of six cows maintained at 1.5 LSUs/ha (LD) and were moved to a grazing area that had a density of 128 LSUs/ha (HD).

	Temperature		Heart rate		Activity	
	LD (*n* = 6)	HD (*n* = 6)	LD (*n* = 6)	HD (*n* = 6)	LD (*n* = 6)	HD (*n* = 6)
MESOR	37.98 ± 1.40	38.02 ± 2.38	69.12 ± 3.85	65.91 ± 2.67	49.39 ± 1.77	40.41 ± 7.23
Amplitude	0.28 ± 0.01	0.28 ± 0.04	4.12 ± 0.88	3.14 ± 0.71	18.14 ± 3.52	11.28 ± 2.67
Acrophase	20:45 ± 0.74^a^	22:45 ± 0.24^b^	19:51 ± 1.05^a^	16:49 ± 1.37^b^	14:47 ± 0.70^a^	12:49 ± 0.46^b^

a, b indicate *p* < 0.05.

## Discussion

The experiment demonstrated that an increase in grazing density induced changes in the circadian rhythmicity of T, HR, and ACT in cows, delaying T acrophase and advancing HR and ACT acrophases. Previous studies described environmentally induced changes in the acrophase of several metabolic or physiological variables in cows. In Holstein cattle, acrophases of the daily rhythms of acute phase proteins were about 2 h later in July than they were in January (Giannetto et al., 2012). In another study, differences affected daily rhythms in blood urea and ammonia concentrations; specifically, cows fed at 16:00 h exhibited nocturnal acrophases in urea, and cows fed at 08:00 or 16:00 h, exhibited diurnal acrophases in ammonia (Piccione et al., 2007). Those authors suggested that external stimuli, such as feeding time, have a direct effect on the rhythms of the metabolites involved in liver function, and on the hypothetic interaction between circadian clocks located in the liver and the mean circadian system. A large experiment involving horses, sheep, goats, and cattle, that were housed in 1-hectare paddocks or in boxes, demonstrated that stable groups of cows and horses experienced an earlier acrophase of the rhythm of ACT (Giannetto et al., 2018), which occurred in our experiment, where HD induced an advance in the acrophase of activity in the cows. Thus, the response of the animals at HD grazing conditions in our experiment were similar to the response of animals in stabled conditions, which exhibited a diminution of their locomotor activity.

Like any circadian rhythm, the rhythm of body T is characterized mainly by its period, which in the absence of external temporal cues is usually very close to 24 h (Refinetti and Menaker, 1992). Body T in cattle has a pronounced circadian rhythm, with a minimum in the morning and a maximum in the late afternoon (Kendall and Webster, 2009), although the circadian rhythm of body T in dairy cows is dynamic and is affected by the physiological state of the animal and environmental stressors. In ruminants, stressors cause a short-lived increase in core body T, i.e., hyperthermia (Lambert and Carder, 2017) by stimulating the sympathetic pathway of the autonomic nervous system and altering the thermoregulatory set point (Pedernera-Romano et al., 2010). Although it was not supported statistically, a pattern of increased in body temperature at night in our experiment may have reflected such hyperthermia related to social stress. It is also likely that the slight body temperature increase at night during HD be a consequence of sleeping/resting more closely together, due to a smaller grazing area. In a diurnal wild species, the tree shrew, subjected to socially induced stress (Fuchs and Flügge, 2002), males were exposed to a dominant male for about 1 h daily, and this social stress increased the mean nocturnal temperature by 0.37°C, which was an emotional fever. Increases in nocturnal temperature occur in humans who are experiencing depression (Souêtre et al., 1988). The cows in our experiment probably experienced higher inter-cow competition at HD than they did at LD, and experienced a higher nocturnal temperature, which may be a reflection of stress. In addition, competition for food can cause stress. For instance, in a study in which mice given a small piece of cheese in the presence of a cage mate who was given a large piece, mice exhibited a large increase in surface body temperature (Watanabe, 2017). Circadian variations in HR and arterial blood pressure in farm animal species is well known (Piccione et al., 2005), and HR has been used as a measure of autonomic regulation of cardiac activity in farm animals to assess stress and well-being under various housing and management conditions (von Borell et al., 2007). Furthermore, changes in HR are a possible early indicator of metabolic stress in cows (Erdmann et al., 2018). The circadian rhythm in activity is disrupted under chronic stress in animals and humans, and it can be less marked if the animal is diseased. For instance, the average level of ACT of a cow on a given day and variations throughout the day differed among specific states such as estrus, lameness, mastitis, and circadian variations in activity appeared to be particularly sensitive and changed 1–2 days before the farmer detected a disorder (Veissier et al., 1989). Probably, grazing density has been a disruption that can modify the circadian rhythmicity of the physiological variables under study.

Although, the light-dark cycle caused by the earth’s rotation is the most important synchronizing agent of circadian rhythms in mammals, a variety of nonphotic factors, such as social cues, can affect circadian rhythmicity (Piccione and Caola, 2002). In hamsters, social interactions, cage cleaning, novelty-induced activity, and immobilization can shift the phase of free-running activity rhythms (Mrosovsky et al., 1989; Van Reeth et al., 1991). Rats subjected to social defeat exhibited pronounced changes in daily rhythms in body T and ACT. After defeat, the amplitude of the T rhythm was strongly reduced, mainly because of an increase in T in the circadian resting phase, but returned to baseline levels after 5–10 days. Group size influences the behavior of grazing cows, including feeding and aggression. Rind and Phillips (1999) observed differences in time spent in, e.g., ruminating and aggression, and the distance from their nearest neighbor among groups of cows that contained four, eight, or 16 animals. Inter-cow competition and walking rate, while grazing were highest in the largest group, and animals in this group spent more time grooming, which often is a displacement activity, which might indicate increased stress. Apparently, regrouping and increases in stocking density have negative effects on cows, and the effects of regrouping are aggravated by concurrent increases in density, and by changes in group and pen size (Talebi et al., 2014). In sheep, ewes in small flocks spent less time grazing than did ewes in large flocks (Penning et al., 1993).

Ruminants maintained in extensive grazing systems have 3–5 grazing events, with the most intense occurring in the morning and in late afternoon/early evening (Gregorini, 2012). At night, they exhibit less intense grazing events, which are 10–15% of daily grazing time. In our experiment, ACT was lowest at night, and cows had 2–4 periods of high movement in the day, although the timing was influenced by grazing density. The absence of differences in activity between grazing densities in the day, and the significant interaction between grazing density and the timing of the peak in the three variables suggest that the change to a higher animal density influenced not only the behavior of the cows in the day and at night, but also the relationship between physiological variables such as T and HR.

## Conclusion

In conclusion, our study suggests that a high animal grazing density might exacerbate the competition for valuable resources for animals, resulting in shifting the circadian rhythmicity of temperature, heart rate, and activity of the cows, advancing or delaying their acrophases. The results of this study highlighted the ability of these bio-loggers to monitor some physiological variables of cows, discriminating changes when animals are managed at different grazing densities.

## Data Availability Statement

The raw data supporting the conclusions of this article will be made available by the authors, without undue reservation.

## Ethics Statement

The animal study was reviewed and approved by the Ethics Committee for Animal Experiments at the University of Salamanca. Written informed consent was obtained from the owners for the participation of their animals in this study.

## Author Contributions

All authors listed have made a substantial, direct and intellectual contribution to the work, and approved it for publication.

## Conflict of Interest

The authors declare that the research was conducted in the absence of any commercial or financial relationships that could be construed as a potential conflict of interest.

## Publisher’s Note

All claims expressed in this article are solely those of the authors and do not necessarily represent those of their affiliated organizations, or those of the publisher, the editors and the reviewers. Any product that may be evaluated in this article, or claim that may be made by its manufacturer, is not guaranteed or endorsed by the publisher.

## Footnotes

^1^ https://cosinor.online
